# Family Partner: study protocol for a pilot randomised study of a home-visitation intervention in Norway

**DOI:** 10.1177/14034948231189773

**Published:** 2023-08-14

**Authors:** Eirin Pedersen, Ira Malmberg-Heimonen, Joakim Finne, Maiken Pontoppidan, Jacinthe Dion, Truls Tømmerås, Anne Grete Tøge

**Affiliations:** 1 Oslo Metropolitan University, Norway; 2 VIVE: the Danish Centre for Social Science Research, Denmark; 3 Université du Québec à Chicoutimi, Canada; 4 The Norwegian Centre for Child Behavioural Development (NUBU), Norway

**Keywords:** child maltreatment, child welfare, feasibility study, intervention, home visitation

## Abstract

**Aim:**

Every year, about 5% of children in Norway experience severe child maltreatment and need support from the child welfare services. However, research-supported interventions for this group are lacking. The current study piloted an intensive home-visitation intervention, Family Partner, which aims to reduce child maltreatment among at-risk parents by improving parental skills, agency and trust in the welfare services, and children’s well-being. The randomised controlled trial piloted in this study examines the acceptability of the Family Partner intervention for staff and families and evaluates its feasibility for a full-scale randomised controlled trial.

**Methods:**

This protocol outlines a prospective, parallel, pilot randomised trial of the Family Partner intervention in three Norwegian municipal child welfare services. The participants are families with children under 12 years of age, where the parents are identified as having challenges. Families in the treatment group receive the Family Partner intervention, while families in the control group receive ordinary child welfare services. Data are collected at baseline, and at 3, 6, 12 and 18 months after recruitment. The pilot study monitor retention and adherence to inform the feasibility of a future full-scale randomised study. To assess the acceptability of the trial and intervention, a subsample of the participating families, as well as the family partners and representatives of the child welfare services in each municipality, are invited to complete qualitative interviews.

**Conclusions:**

**The results will guide the design of a fully powered randomised controlled trial of the Family Partner intervention compared with ordinary child welfare services.**

**Trial registration:**

ClinicalTrials.gov identifier: NCT04957394; Pilot Trial of Family Partner: a Child Maltreatment Prevention Intervention (FAMPART); registered on 12 July 2021.

## Background

Estimates suggest that every year, about 5% of children in Norway are subject to severe child maltreatment, that is, physical or emotional violence and neglect [1,2]. Physical neglect occurs when a caregiver fails to attend to the child’s basic physical needs, such as providing sufficient food, healthcare and clothing. Emotional neglect refers to situations where the caregiver fails to meet the child’s psychological needs, such as providing adequate love, care, attention and stimulation [3]. Children experiencing maltreatment often need support from the Child Welfare Services (CWS), but only 2.5% of children and families receiving CWS in Norway have access to interventions that have demonstrated being effective based on scientific research [4]. While several parenting interventions, such as the Circle of Security, Tuning Into Kids, Parent Management Training Oregon and The Incredible Years have been implemented and evaluated in Norway [5], it is among one of few European countries that has not established largescale implementation of home-visitation interventions [6].

Responding to the CWS’s need for research-supported interventions, this study piloted a new home-visitation intervention, the Family Partner. The intervention aims to reduce child maltreatment and promote healthy development and has been specifically developed for Norwegian parents’ and children’s needs. It was implemented in three municipal CWS across Norway. The aim of the pilot study is to examine the acceptability of the Family Partner intervention for staff and families and evaluate its feasibility for a full-scale randomised controlled trial (RCT).

### Previous research on child maltreatment

Experiencing child maltreatment predicts adverse trajectories that are difficult to reverse later in life [7]. This potentially includes an elevated risk of fatal injury, adverse health outcomes and reduced opportunities for a fulfilling life [7,8]. Studies conducted among survivors of child maltreatment suggest elevated risks of a multitude of social and health problems [7], including mental health problems and behavioural problems [9] and physical health issues [10].

Systematic reviews indicate that high-quality parental training and home-visitation interventions can improve parenting practice and behaviour [11,12] and reduce future child maltreatment [13]. Accordingly, some meta-analytic reviews have identified how certain characteristics of child maltreatment interventions are associated with their effectiveness. Within this strand of research, the meta-analyses by van der Put [14] found that interventions with the potential to address existing cases of child maltreatment are more effective at reducing its occurrence than interventions aimed solely at prevention. Within the category of preventative interventions, van der Put [14] found that shorter interventions (0–6 months) were most effective. Further, larger-effect sizes were found for child maltreatment interventions focusing on parental self-efficacy and skills. Chen and Chan [8] demonstrated that improving parenting skills is essential for the effectiveness of interventions aiming to reduce child maltreatment. In the context of home-visitation programmes, Gubbels [15] demonstrated stronger effects from interventions that focused on improving parental expectations of the child or parenthood, and interventions targeting parental responsiveness to the child’s needs.

Child maltreatment has been measured in various ways in previous research, including self-report of actual or potential child abuse among caregivers through, for example, the Child Abuse Potential Inventory [16] and the Conflict Tactics Scale [17]. Other measures include retrospective self-report of own childhood, for example, the Adverse Childhood Experiences questionnaire [18] and the Childhood Trauma questionnaire [19]. Some studies investigating effects of preventative interventions also use emergency attendances or referral to out-of-home care or reports to the CWS as proxies for child maltreatment [20,21], where the hypothesis is that the incidence will be reduced if child maltreatment is prevented. However, studies assessing interventions aimed at reducing child maltreatment could also measure more long-term or indirect outcomes, such as criminal and antisocial behaviour [22,23].

### Family Partner intervention

The Family Partner intervention is an intensive home-visitation family intervention that aims to reduce child maltreatment among at-risk parents by improving parental skills, agency and trust in the welfare services, and children’s well-being. The intervention is being delivered by child welfare workers employed by the Norwegian CWS. These workers typically hold a 3-year bachelor’s degree in social work or an equivalent qualification. During the study, the child welfare workers receive implementation support, such as training and supervision in the Family Partner methodology.

The Family Partner intervention procedures include: (a) parental training; (b) home visitations; (c) practical assistance; (d) a measurement feedback system including monthly well-being scoring schemes; (e) an emphasis on a therapeutic relationship with parents to create trust; and (f) coordination of services. Central to the intervention is the family partner, who is a professional employed to ensure that the families receive close, empowering and structured support.

The intervention is based on three theoretical perspectives: Banduras’s [24] theory of self-efficacy, which states that your belief in your abilities impacts your chance of succeeding; Bronfenbrenner’s [25] ecological theory of the family as a context for human development; and Ablon’s [26] theory of the potential of a collaborative problem-solving approach.

The estimated duration of the intervention is 9 months, but it can be extended by 3 months if considered beneficial to the families. The intervention period is divided into three phases: a 3-month start-up phase, a 3–6-month working phase and a 3-month ending phase. During the intervention period, the family partners provide each family with an average of 5 hours of weekly service, with more frequent service at the beginning and less frequent during the ending phase. In total, the number of sessions with each family across the three periods should be between 50 and 100. It should be noted the intervention may be modified based on findings from the pilot study, including assessments provided by the managers of the municipal agencies, family partners, parents and children.

## Design and methods

### Trial design

The study is a prospective, parallel, pilot randomised trial of the Family Partner intervention. Families in the treatment group receive the Family Partner intervention, while families in the control group receive ordinary child welfare services (e.g., guidance and advice for the family, parent groups or home-based assistance).

After consenting to take part in the study, one of the primary caregivers completes the baseline assessment questionnaire and is then randomised 1:1 to the treatment and control groups.

The recruitment and randomisation of families is an ongoing process. Each family partner can serve up to five families at a time, and each family participate for up to 12 months. When a family leaves the intervention, a new family is recruited and randomised.

**Figure 1.**
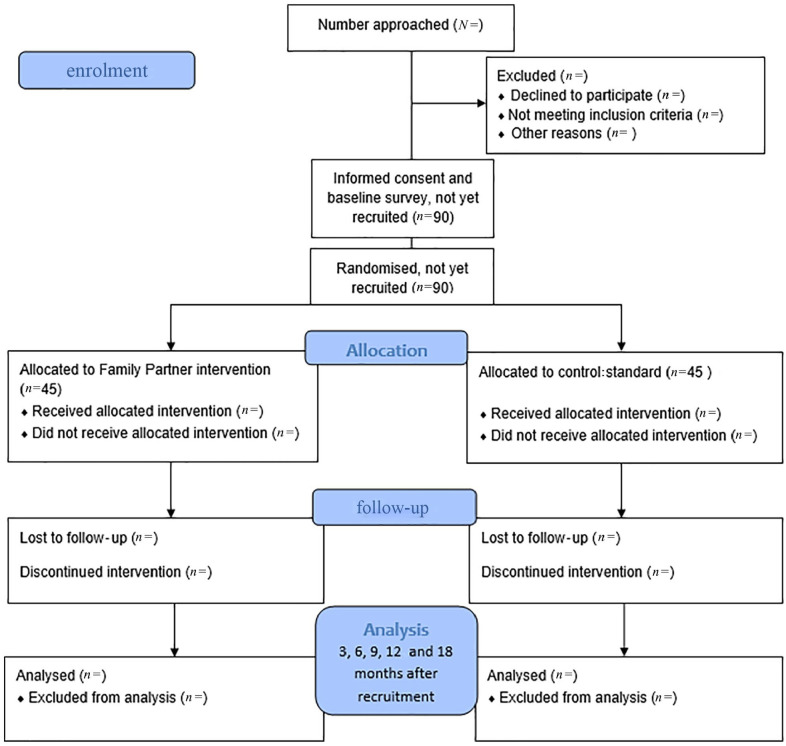
Study flow diagram.

The trial is piloted at three sites; two of which are in medium-sized cities and one in a sparsely populated municipality. These sites were included in the pilot based on their motivation for participation, and because they vary in size and location. A flow diagram of the study design is provided in Figure 1.

We monitor and assess implementation. After each visit or interaction with a family, the family partners take a digital survey containing questions on the length of the visit, whether it was a physical meeting or a phone call/text message, where the meeting took place, who was present at the visit and the theme of the visit. The information obtained provides an overview of the intervention’s delivery and how it develops over the three stages. The family partners receive an overview of their registered visits once a month.

### Participants

The participants in this pilot trial are families with children. The inclusion criteria for recruitment are families where:

(1) the parents have at least one child under the age of 12;
(2) the parents have challenges regarding parental skills, that is, routines, boundaries, upbringing methods, emotional attachment to the child, understanding the child, monitoring the child’s arenas or practical care abilities (equipment, food, clothes, hygiene).

In terms of the first inclusion criterion, the Norwegian CWS is typically divided into departments depending on the age of the child at risk, with one department for children in the age group 0–12 and one for adolescents above the age of 13. Since the intervention is targeted towards younger children, the cut-off age criterion adheres to the structure of the Norwegian CWS. Social workers in the CWS assess new families and determine whether they fit the inclusion criteria and are able to participate in the study. Participation is voluntary, and all participating families gave written consent. The pilot started recruiting on 2 January 2022 and continued until 2 March 2023.

### Data and outcomes

#### Primary outcomes

The primary outcomes in the pilot trial are acceptability, retention and adherence. In line with Finn and Sladeczek [27], acceptability is defined here as the fairness and expected effectiveness for the end users, that is, the families.

We explore the acceptability of the trial and intervention through qualitative interviews with three types of informants: (a) a subsample of participating families; (b) the family partners; and (c) representatives of the CWS in each municipality. We interview three groups of informants: (a) participating families (*n* = 10); (b) professionals delivering the intervention (*n* = 9); and (c) other services impacted by the intervention (*n* = 6). In line with the evaluation design, we follow the implementation process and interview informants at different timepoints of the intervention. We interview participating families during and after the intervention, while the professionals are interviewed in individual and group interviews four times during the intervention. The other services will be interviewed once towards the end of the intervention. We use semi-structured interview guides for all interviews. The interview guide cover a wide range of themes, such as experiences with recruitment, study design, delivering and implementing the intervention, and beliefs about the intervention’s feasibility. The interviews are recorded and transcribed verbatim. Both the audio files and the transcriptions are stored in a secure location.

We carry out ethnographic fieldwork which consists of visits at three different times to the three implementation sites and observations of the family partners (walk-along method) when they interact with the families. The field work at the implementation sites will give insight into the organisational context of the intervention, as well as challenges, strengths and differences in implementation between the sites. Using the walk-along method with the family partners will provide an understanding of both the relational and practical aspects of the trial and intervention provision. Data from the ethnographic fieldwork consists of fieldnotes and interview data.

We assess retention in the trial by monitoring response rates, that is, whether participants respond to the surveys. We assess adherence to the trial by registering whether families withdraw their consent within 12 months of recruitment. We measure adherence to the intervention by monitoring if, when and why families terminate the intervention.

#### Secondary outcomes

The secondary objective of the pilot trial is to explore changes over time, to get an indication of the potential effects of the intervention on parental abilities and the families’ use of welfare services. This can be used to inform a sample size calculation for a full trial. We measure the effects of the Family Partner intervention on parenting behaviour, parental stress, parent locus of control, family routines, parental mental health and self-efficacy, relations to partner, experience of social support, service use and relationship to the professional helper (see Table I).

**Table I.** List of pilot trial objectives, evaluation measures and timepoints.

What	Instrument	Timepoints (months since baseline)						
		Baseline	3	6	9	12	18	Number of items
**Primary objectives**								
Participant acceptability	Qualitative interviews with participating families, the family partners, and representatives of the Child Welfare Service (CWS) in each municipality							
Retention in the study	Comparison of response rates, that is, how many participants respond to the surveys, by study arm							
Adherence	Monitoring the share of families who terminate the intervention within 3 months of enrolment							
**Secondary objectives**								
Parenting behaviour	Parenting Scale Short Form (PS-8)			x	x	x	x	8
Parental stress	Parental Stress Scale (PSS)	x				x	x	18
Parent locus of control	Parent Locus of Control (PLoC)		x		x			5
Family routines	Developed by Pontoppidan, based on the Child Routine Inventory	x		x				3
Behavioural screening	Strengths and Difficulties Questionnaire (SDQ-25)		x					34
Parental mental health	The Warwick–Edinburgh Mental Well-being Scales (WEMWBS)		x		x	x	x	14
Self-efficacy	The General Self-efficacy Scale (GSE)	x	x	x	x	x	x	10
Social support	Oslo Social Support Scale (OSS-3)			x		x	x	3
Relationship to professional helper	Working Alliance Inventory (WAI)	x	x	x	x	x	x	12
Service use	Developed by Malmberg-Heimonen, Tøge and Pedersen				x	x	x	5
Relations to partner	Parenting and Family Adjustment Scales (PAFAS)			x				4
Parent’s own childhood	Developed by Tøge and Pedersen	x						2

Parenting behaviour is measured through the Parenting Scale Short Form (PS-8), an eight-item validated scale measuring parenting *overreactivity* and *laxness* [28]. Parenting stress is measured through the revised Norwegian version of the 18-item Parenting Stress Scale (PSS) [29]. Parents’ perceived power and efficacy in child-rearing situations is measured through five items (2, 4, 39, 40 and 43) from the Parental Control of Child’s Behaviour subscale of the Parent Locus of Control (PLoC) [30]. We use five items from the Daily Living Routines subscale and the Household Responsibilities subscale of the Child Routine Inventory (CRI: a 39-item version). Inspired by the CRI, we developed five additional items on family routines relating to mealtimes and language. Mental well-being in adults is measured through the Warwick–Edinburgh Mental Well-being Scale (WEMWBS), which has been validated in a Norwegian context [31]. The Strengths and Difficulties Questionnaire (SDQ-25) is used to measure prosocial behaviour and psychopathology for children between the age of 4 and 17 and has been validated in several studies [32]. We used the Norwegian version of the General Self-efficacy Scale (GSE) to measure the belief one has in one’s own ability to solve challenges [33]. Social support is measured through the Oslo Social Support Scale (OSS-3) [34]. We used the Working Alliance Inventory (WAI-SR) as a measure of the working alliance between the helper and helpee [35]. To assess service use, we ask parents to report how many times the family has been in contact with their general practitioner, the specialist health service (e.g., hospital), educational psychology services, the Norwegian Labour and Welfare Administration and the child and adolescent psychiatry during the previous month. To address the ‘relations to partner’ objective, we use three items on parental teamwork from the Parenting and Family Adjustment Scales (PAFAS) [36]. At baseline, we assess the parents’ childhood experience with the CWS by asking them whether they or their parents have at any time been in contact with the CWS. If yes, we ask them how appropriate the service was (using a five-point Likert scale). If no, we ask them whether they think that their parents should, at some point, have received help from the CWS (yes or no).

We had originally intended to include all the measures presented in Table I in the baseline questionnaire. However, after the first families had been recruited, the family partners at one of the sites felt that the questionnaire was too long to complete. Based on this feedback, it was reduced from twelve to five measures before the recruitment was continued. During the pilot period, we monitor the time spent on responding to the questionnaire. If most families spend less than 20 minutes, we will consider it possible to expand the baseline survey in a future RCT. Any changes to the intervention and manual after the pilot trial commencement will be reported along with the study results.

Beyond survey data, Norway has rich administrative data that track societal and health-related aspects of the population. By utilising administrative data from the municipal CWS, we can evaluate the impact on various aspects, including the number of concerns submitted to CWS, the assistance measures implemented by CWS and the number of care takeovers.

#### Sample size

The study aim to include 90 families, 30 from each of the sites. This number is expected to be sufficient to inspect randomisation routines, acceptability, retention and adherence. Contrary to a potential full-scale RCT, the pilot study does not aim to estimate effect sizes. However, if the retention is 80%, and we apply conventional power calculation assumptions (*α* = 0.05 and power set to 80%), our sample (*N* = 90) would be sufficient to detect a medium-to-large effect (*d* = 0.67) on continuous outcomes.

#### Randomisation

Families that fulfil the inclusion criteria are invited by social workers in the CWS to take part in the study. After consent, one of the parents complete the baseline survey (https://nettskjema.no/). As a crucial component of the digital survey, families are assigned randomly to either the intervention or control group. The survey has an integrated ‘toss coin’ function, it randomly generates one out of two numbers, either 240025 or 240197. This number is available to the researchers but concealed for the responding parent. Once a parent completes the baseline survey, the researchers extract the assigned number and allocate the family to the intervention or control group accordingly. Specifically, participants with the number 240025 are assigned to the treatment group, where they receive the Family Partner intervention, while those with the number 240197 are assigned to the control group, where they receive treatment as usual. The allocation ratio for the randomisation is 1:1, meaning that participants have a 50% chance of being randomised to the intervention or control group. After randomisation, the research team is immediately notified by email, and informs the CWS of where the participant has been allocated.

#### Analyses

We will apply qualitative and quantitative methods to identify factors affecting trial feasibility. Further, we will assess the feasibility of the intervention and provide knowledge that can be used to explore indications of an inherently faulty concept or theory or implementation failure; see, for example, the work by Malmberg-Heimonen and Tøge [37].

We will apply a thematic analytical strategy to both interviews and observations, using themes such as, for example, ‘randomisation experiences’, ‘relationship between families and professional helpers’, ‘organisational boosters and barriers to implementation’ and ‘contribution of the intervention to existing services’.

Retention to the trial will be analysed by investigating the response rates. At each timepoint for survey data, we will calculate the pooled response rate and use a chi-squared test to check whether response rates differ by study arm.

To assess adherence to the trial, we will calculate the rate of families who withdrew their consent within 12 months of recruitment and use a chi-squared test to check whether adherence to the trial differs by study arm. To assess adherence to the intervention, we will calculate the rate of families who terminate the intervention within the first 3 months after enrolment.

The secondary outcomes will be analysed according to conventional procedures for RCTs. Using survey data and administrative data from the municipal CWS, we will examine records of child maltreatment, child behaviour, parenting behaviour, parental stress, parent locus of control, family routines, parental mental health, self-efficacy, social support, relations to professional helper, relations to partner and service use. All analyses and results will be conducted and presented according to recognised academic standards [38], including adherence to the intention-to-treat principle, where all consenting participants are included in the analyses. For binary outcomes, we will apply logistical models and report effect sizes as odds ratios. For continuous outcomes, we will use linear models and report coefficients as standardised effect sizes (Cohen’s *d*). The analyses will be adjusted for observed differences at baseline and repeated hypothesis tests.

#### Success criteria

We will consider the pilot trial a success if we achieve the following goals:

(1) 70% of the participants complete their allocated treatment (at least 9 months) and this is equal in both groups (*p* > 0.05);
(2) response rates are above 70% and are equal in both groups (*p* > 0.05);
(3) all sites are able to recruit the necessary staff, retain at least 50% of family partners for at least 18 months, and are able to recruit new staff in the event of turnover.

If the trial is successful, we will report estimated costs for an upscaled trial.

#### Ethical considerations

To safeguard ethical considerations, we follow the guidelines of the Norwegian National Research Ethics Committees. The study has been assessed and approved by the Norwegian Centre for Research Data (reference number 804402) and registered at ClinicalTrials.gov (NCT04957394). Participation is based on parental consent, and all participants can withdraw from the study at any time and for any reason.

The researchers are subject to professional confidentiality in relation to all data and analyses. All data collections will follow consent procedures according to guidelines and advice from the Norwegian Centre for Research Data and the Norwegian Data Protection Authority. The survey data are collected through an electronic survey system and stored and managed through Services for Sensitive Data (TSD), a secure server with a two-step authentication process. All data processing, including coding and analyses, is conducted in TSD.

#### Long-term follow up

To facilitate long-term follow up, we will request permission to contact the families at a later date during the final survey. Given the small scale of the study, it is possible that we may not have adequate statistical power to conduct a thorough analysis. Nonetheless, establishing contact with the families and conducting qualitative interviews could provide valuable insights into the longer-term outcomes for these families.

## Discussion

This feasibility study allow us to investigate the acceptability of the Family Partner intervention for staff and families and evaluate the feasibility of a full-scale RCT of the intervention. If rates of recruitment, retention and response are adequate, and the study procedures are found to be acceptable, we will consider conducting a full trial. As this is a pilot feasibility trial, it is not powered to estimate the effects of the intervention. In addition, there are further limitations associated with treatment contrast in this study, that is, the comparison between the Family Partner intervention and the control group.

The organisation of municipal services in Norway, specifically CWS, varies across different locations, resulting in varying access to resources and services. Consequently, CWS is also organised differently across municipalities, resulting in differing service offerings. Furthermore, there is limited practical integration between specialist health services, such as district psychiatric centres, municipal mental health services and CWS. This lack of collaboration makes it difficult to predict the services the control group would receive. Additionally, there are considerable differences in the educational backgrounds of child welfare workers in various geographic locations, which could potentially create disparities in the results when comparing different sites. Hence, the research group will have to carefully assess the services provided to the control group.

Results from the pilot will help to assess the acceptability of the intervention’s implementation and delivery among frontline practitioners in the CWS. This information will be crucial to understand whether the intervention is a suitable fit for the Norwegian child welfare infrastructure and local services.
